# The effect of trait anxiety on attentional mechanisms in combined context and cue conditioning and extinction learning

**DOI:** 10.1038/s41598-019-45239-3

**Published:** 2019-06-20

**Authors:** Yannik Stegmann, Philipp Reicherts, Marta Andreatta, Paul Pauli, Matthias J. Wieser

**Affiliations:** 1 Department of Psychology (Biological Psychology, Clinical Psychology, and Psychotherapy), University of Würzburg, Würzburg, Germany; 2 Center of Mental Health, University of Würzburg, Würzburg, Germany; 3 Department of Psychology, Education, and Child Studies, Erasmus University Rotterdam, Rotterdam, The Netherlands

**Keywords:** Attention, Fear conditioning

## Abstract

Sensory processing and attention allocation are shaped by threat, but the role of trait-anxiety in sensory processing as a function of threat predictability remains incompletely understood. Therefore, we measured steady-state visual evoked potentials (ssVEPs) as an index of sensory processing of predictable and unpredictable threat cues in 29 low (LA) and 29 high (HA) trait-anxious participants during a modified NPU-paradigm followed by an extinction phase. Three different contextual cues indicated safety (N), predictable (P) or unpredictable threat (U), while foreground cues signalled shocks in the P-condition only. All participants allocated increased attentional resources to the central P-threat cue, replicating previous findings. Importantly, LA individuals exhibited larger ssVEP amplitudes to contextual threat (U and P) than to contextual safety cues, while HA individuals did not differentiate among contextual cues in general. Further, HA exhibited higher aversive ratings of all contexts compared to LA. These results suggest that high trait-anxious individuals might be worse at discriminating contextual threat stimuli and accordingly overestimate the probability and aversiveness of unpredictable threat. These findings support the notion of aberrant sensory processing of unpredictable threat in anxiety disorders, as this processing pattern is already evident in individuals at risk of these disorders.

## Introduction

Attention to threat facilitates adaptive behaviour in changing environments and is therefore a crucial mechanism for improving survival in potentially hazardous situations^1^. Threat may either be specific and highly predictable or diffuse and non-predictable^2^. Likewise, it has been suggested, that threat predictability plays a major role in the differentiation of fear and anxiety^3^, though its underlying mechanisms are still under debate^4,5^. In the DSM-5, fear is defined as the emotional response following real or perceived imminent threat, while anxiety is described by the anticipation of unspecific, future threat^6^. For this reason, anxiety can be characterized by persistent hypervigilance as a response to potential danger in ambiguous situations whereas during fear, attention allocation becomes more selective as threat becomes more imminent^2,7,8^. Richards *et al*.^9^ suggest that these attentional processing styles serve different functions: hypervigilance is associated with a broadening of attention to facilitate threat detection, which is crucial for identifying danger in an ambiguous environment; in contrast, selective attention is accompanied by a narrowing of attention onto the threat stimulus to ensure preferential processing and consequently improve impending fight-or-flight reactions.

Aversive cue conditioning paradigms have been used to experimentally induce predictable threat: discrete visual stimuli (conditioned stimuli, CS) predict upcoming aversive events (unconditioned stimuli, US), modelling aspects of acute (phobic) fear responses^10,11^. In contrast, one way to induce unpredictable threat is by means of context conditioning, where USs are presented during a context (e.g. a virtual office) in the absence of any discrete cues^12^. Consequently, the conditioned context (CTX) in which individuals experience the aversive events becomes the best predictor leading to a sustained state of anxious apprehension while being in that context^13,14^. Eventually, extinction learning takes place, when the former conditioned cue or context is repeatedly presented unreinforced and consequently conditioned fear or anxiety responses are gradually reduced^15^. Importantly, extinction learning is discussed as one of the main mechanisms underlying exposure therapy^16^.

In human research, fear and anxiety are often compared by using the NPU-threat task^17^. The original version of the NPU-threat task consists of three conditions (i.e., contexts): two threat conditions, in which aversive events are administered either predictably (P) or unpredictably (U), and a neutral condition (N), during with no aversive events. Each condition is indicated by a different verbal instruction and contains up to three short presentations of a centrally presented cue. However, the central cue reliably announces the presentation of aversive events in the P-condition only, while aversive events are presented unpredictable in the U-condition. Typically, participants become fully informed about the US-contingencies before the experiment. In previous studies, startle eye blink reflex is recorded as an index of fear and anxiety^18^. Several studies demonstrated fear-potentiated startle responses during cue presentations in the P-condition and anxiety-potentiated startle responses during the U-condition^18–20^.

Numerous studies using the NPU-threat task suggest that heightened reactivity to unpredictable, but not predictable threat is characteristic for anxiety disorders, e.g., panic disorders^21^, PTSD^22^, social anxiety disorders and specific phobias^23^. Therefore, exaggerated responding to unpredictable threat is discussed as a potential biomarker for disorders associated with symptoms of fear and anxiety. To further investigate the role of hypersensitivity to unpredictable threat in the development of anxiety disorders, it is important to examine individuals at risk of these disorders. Since elevated trait-anxiety is an important risk factor for anxiety disorders^1,24^, one way to achieve this goal is to compare high trait-anxious participants not suffering from clinical disorders with average or low trait-anxious participants.

However, findings on the influence of trait-anxiety on aversive conditioning are mixed. For example, trait-anxiety measured by the Spielberger State-Trait Anxiety Inventory (STAI)^25^ did not correlate with discriminatory fear learning in a differential aversive conditioning paradigm^26^. Nevertheless, recent studies reported that trait-anxiety is positively associated with threat responding when threat stimuli are uncertain^12,27,28^, which is in line with the hypothesis of heightened reactivity to unpredictable threat in anxious individuals.

Recently, attentional mechanisms in aversive conditioning and extinction learning have been elucidated by using steady-state visual evoked potentials (ssVEPs). SsVEPs are oscillatory, electrocortical responses to periodically flickering visual stimuli, whose frequency equals that of the driving stimuli^29^. Recorded by electroencephalogram (EEG), the ssVEP signal can be transformed into the frequency-domain, where the amplitude of the driving frequency is a marker of early stages of visual processing and attention allocation. Various studies demonstrated that the ssVEP amplitude is sensitive to affective and attentional features like emotional picture content^30^, motivational significance^31^ or the presence of attended compared to unattended features^32^. Since the underlying frequency is well known, the ssVEP signal can be accurately separated from background noise, resulting in an excellent signal-to-noise ratio, that is robust to eye blink and movement artefacts^33,34^. In differential aversive conditioning paradigms, the threat-predicting cue (i.e., CS+) reliably leads to increased ssVEP amplitudes compared to the stimulus not associated to the threat (i.e., CS-)^35–39^. It is assumed that changes in visuocortical activation during aversive learning reflect re-entrant modulations of the visual system from higher-order cortices to increase perceptual sensitivity^40,41^. For instance, McTeague *et al*.^42^ used an instructed aversive conditioning paradigm, in which a series of differently oriented visual cues served as conditioned stimuli. Results demonstrated enhanced response amplitudes for the CS+ compared to CS− during acquisition. Moreover, ssVEP amplitudes to stimuli with the greatest similarity to the CS+ were increasingly suppressed. During extinction, an inverted pattern was shown, i.e., CS+ features suppressed ssVEP amplitudes, while CS− stimuli enhanced response amplitudes^42^. This pattern already appeared after three trials of extinction, suggesting that visuocortical activity is influenced by top-down influences, which may reflect reorientation of attention after the instructed change of contingencies. In a recent ssVEP study, Ahrens *et al*.^43^ used ssVEPs in a differential conditioning paradigm with socially conditioned stimuli to compare low socially anxious with high socially anxious individuals. Results showed that only low socially anxious individuals differentiated between conditioned stimuli suggesting that high socially anxious individuals are characterized by an impaired ability to discriminate between CS+ and CS- compared to low socially anxious individuals.

Most importantly, ssVEPs can be used to independently quantify attention allocation to two or more competing visual stimuli presented at the same time, if they flicker at different frequencies (frequency tagging; e.g.^44^). Therefore, ssVEPs constitute an ideal tool to simultaneously record the strength of visual processing of e.g. central and peripheral (contextual) cues in a combined conditioning paradigm^45^. In a recent study, our lab used this technique in an adapted NPU-threat task to quantify electrocortical correlates of attention allocation during a combined cue and context conditioning paradigm^7^. We demonstrated hypervigilance to the onset of unpredictable threat contexts and increased visuocortical processing of the predictable threat stimulus. However, no study has yet examined the influences of trait anxiety on attentional processing of predictable and unpredictable threat during acquisition and extinction learning.

The aim of the present study was to compare high and low anxious individuals with the NPU-threat task regarding the allocation of attentional resources as indicated by ssVEP during threat acquisition and extinction. According to previous results, we tested whether visuocortical processing of predictable and unpredictable threat cues is aberrant in high compared to low anxious individuals. Furthermore, we predicted reduced extinction learning in high vs. low anxious individuals^46^.

## Material and Methods

### Participants

The sample included sixty subjects recruited via advertisements on the internet and on a local participant-platform. Participants were required to be between 18 and 35 years old, free of any family history of photic epilepsy, any psychiatric or neurological disorders, and have normal or corrected vision. All participants completed a pre-screening including four items of the STAI-Trait questionnaire, which showed the highest correlation with the STAI-Trait sum-score^47^ in a large screening sample (n = 526), that was recruited within the context of the Collaborative Research Centre (SFB-TRR58) at the University of Würzburg (for further details see^48^). On this basis, we used a regression approach to estimate the STAI-Trait score from four items of the STAI-Trait questionnaire that explained the most variance of the total scores to select 30 high and 30 low trait-anxious participants. Cut-offs were chosen based on the distribution of the local sample. The resulting cut-off scores were ≤33 (≤40%-quantile) for low trait-anxious and ≥41 (≥80%-quantile) for high trait-anxious participants. All participants completed the entire German version of the STAI-Trait questionnaire during the experimental session (see Table 1). The classification was mainly confirmed, only one participant of each group fulfilled the criterion of the respective opposite group and was consequently excluded, resulting in a final sample of 29 high trait-anxious (15 females) and 29 low trait-anxious (15 females) participants. To further characterize the final sample, participants were required to complete several questionnaires measuring symptoms of anxiety, including the Anxiety Sensitivity Index 3^49,50^, Intolerance of Uncertainty Scale^51,52^ and the GAD-7^53^. All participants gave written informed consent and were paid 15 € or received an equivalent in course credits. The experiment was performed in accordance with relevant guidelines and regulations. All procedures were approved by the ethics committee of the University of Würzburg.

**Table 1:** Questionnaire and sociodemographic characteristics of the low and high anxious group.

Group: Variable	Low Anxious (n = 29, 15 f)		High Anxious (n = 29, 15 f)		*t*-test		*α*
	*M*	*SD*	*M*	*SD*	*t*(56)	*p*	
STAI – Trait	29.48	4.57	47.41	7.23	11.29	<0.001*	0.94
STAI - State	33.03	6.04	38.31	5.21	3.56	<0.001*	0.83
ASI-3	13.45	10.80	22.07	10.96	3.02	=0.002*	0.92
IUS	37.62	9.11	53.21	10.18	6.15	<0.001*	0.90
GAD7	2.93	2.15	7.31	3.38	5.88	<0.001*	0.81
Age	24.62	3.54	25.62	3.41	1.10	=0.278	
Pain threshold [mA]	1.63	1.02	1.43	0.94	0.76	=0.448	

STAI = State-Trait Anxiety Inventory; ASI-3 = Anxiety Sensitivity Index 3; IUS = Intolerance of Uncertainty Scale; GAD-7 = Generalized Anxiety Disorder Screening Scale; *α* = standardised Cronbach’s alpha; *p < 0.05.

### Materials and stimuli

The stimuli used were similar to those described in Wieser *et al*.^7^. Briefly, central cues were black-and-white Gabor patches, consisting of sinusoidal grating (Gaussian-windowed with maximal contrast at centre) with a spatial frequency of 1.4 cycles per degree. From a viewing distance of 100 cm, the Gabor patches spanned a visual angle of 5.6° horizontally and vertically. We used three different orientations (−45°, 0° and 45°) for the three contextual conditions. Visual stimuli were counter-balanced for conditions across participants. Contextual cues were an array of four triangles, squares or circles, presented peripherally ca. 4.7° of visual angle from the central grating, spanning visual angles of 2.4°. All visual stimuli were presented on a 19-inch monitor (resolution = 1024 × 768 pixels) with a vertical refresh rate of 60 Hz, located ca. 80 cm in front of the participant, using the Presentation software (Neurobehavioral Systems, Inc., Albany, CA, USA).

Aversive unconditioned stimuli (US) were 20 ms electric pulse trains, composed of 5 pulses of 2 ms and intervals of 2 ms between the pulses, which were delivered to the left calf through surface bar electrodes consisting of two gold-plated stainless-steel disks of 9 mm diameter and 30 mm spacing. The electric stimuli were generated by a constant current stimulator (Digitimer DS7A, Digitmer Ltd., Welwyn Garden City, UK). Prior to the actual experiment, the US were adjusted to the individual pain-threshold. Thus, participants received two series of increasing and decreasing intensities until they reached a level they described as “just noticeable pain” – corresponding to 4 on a scale from 0 (no pain at all) to 10 (unbearable pain). The individual US intensity was determined by calculating the mean of the four series’ final intensities and then adding 30% to avoid habituation. The resulting intensities and subjective pain ratings were 2.2 ± 1.4 mA (mean ± *SD*) for LA individuals and 1.9 ± 1.2 mA for HA individuals, *t*(56) = 0.91, *p* = 0.366, respectively 5.4 ± 0.8 for LA individuals and 5.7 ± 1.5 for HA individuals, *t*(56) = 0.86, *p* = 0.394.

### Procedure

**Figure 1:**
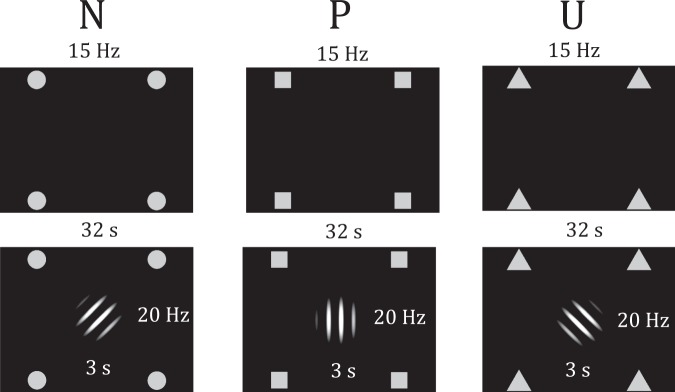
Design. The contextual cues (top row), consisting of 4 peripherally presented geometrical symbols, flickered for 32 s with a frequency of 15 Hz. At random time points, the central cue (bottom row) is presented centrally for 3 s, flickering with a frequency of 20 Hz. The central cue was predictably associated with an aversive stimulus in the P-condition. During the U-condition, aversive stimuli were unpredictably presented independent of the central cue presentation. No US were delivered during the N-condition. The central cues are slightly tilted for each condition to disentangle the predictive meaning of central and contextual cues.

Participants completed the questionnaires before being seated in a sound-attenuated, dimly lit testing room, where the EEG-net was applied. Participants then were instructed to fixate a centrally presented fixation cross and to reduce any movements including eye movements and blinks during the experimental session. We used a modified version of the original NPU-threat task^17^, similarly as reported previously^7^. In brief, three different conditions were indicated by the presentation of the contextual cues for 32 s (one block), flickering at a frequency of 15 Hz (Fig. 1). During each block, two or three Gabor patches were presented centrally for 3 s, flickering at 20 Hz. Each Gabor patch was randomly presented between 6 s and 27 s after context onset, with an inter-stimulus-interval of at least 3 s. In the predictable condition (P), Gabor patch offset was paired with an US presentation (100% reinforcement rate). In the unpredictable condition (U), two or three US were presented unpredictably between 5 s and 30 s after context onset, with a temporal lag of at least 3 s, but never during the presentation of a Gabor patch. In the neutral condition (N), no US were presented. Participants were fully instructed about the US-contingency. To avoid that context onset and central cue presentation during the U-condition acquired safety-signal properties, we implemented a practice phase of 6 blocks (each condition twice) prior to the actual acquisition phase, in which one US in the U-condition was always presented during the first 5 s and another during the central cue presentation. After the practice phase, participants were also familiarized with the rating scales. The acquisition phase consisted of 36 blocks overall (12 per N, P, or U-condition) with half of each block containing two and three central cue presentations respectively, resulting in 30 central cue presentations per condition. In sum, 30 US were also presented during the U-condition to obtain the same amount of US as during the P-condition. Blocks were separated by inter-trial-intervals (ITIs) of random durations between 2,500 ms and 3,500 ms. To investigate extinction learning, we implemented an additional phase, which equalled the acquisition phase in number of blocks and stimulus timing, except no US were delivered. Participants were not instructed about the absence of the US during extinction. After every 12^th^ block, participants were asked to rate the contextual cues and the combination of context and central cue separately regarding perceived threat (“*How threatening do you perceive this picture?*”, NRS, 1 = not threatening at all – 9 = very threatening) and US-contingency (“*To what extent do you expect an electrical stimulus meanwhile this picture is present?*”, VAS, ranging from 0 to 100%).

### EEG recording and data analysis

EEG data analysis was conducted and is reported according to published guidelines^54^. Electrocortical brain activity was recorded using a 129 electrodes Electrical Geodesics System (EGI, Eugene, OR) referenced to the vertex electrode (Cz), with a sampling rate of 250 Hz and an on-line band-pass filter of 0.1–100 Hz. Electrode impedances were kept below 50 kΩ. Subsequent data processing occurred off-line using the EMEGS software for Matlab^55^. In a first step, all data were filtered using a 40-Hz low-pass filter (cut-off at 3 dB point; 45 dB/octave, 19^th^ order Butterworth), before extracting epochs from 600 ms pre- to 2,900 ms post-onset for central cue and 600 ms pre- to 4,900 ms post-onset for contextual cue responses. Following the guidelines for the statistical correction of artefacts in dense array studies procedure (SCADS^56^), we first detected individual channel artefacts based on the original recording reference (Cz), before data were re-recorded to the average reference to identify global artefacts. Bad sensors within individual trials were identified based on rejection criteria for the distributions of the maximum absolute amplitude, standard deviation and gradient. Contaminated trials were removed, if they included more than 20 bad sensors. After rejection, contaminated sensors of the remaining epochs were interpolated using weighted spherical splines fit to all remaining sensors. The mean rejection rate for contextual cue and central cue responses were 28% ± 7 (mean ± *SD*) and 23% ± 10 (mean ± *SD*), respectively. Also, there were no group differences for the total number of usable trials (HA: 193.1 ± 21.1, NA: 188.9 ± 24.2, t(56) = 0.64, p = 0.525) nor for the number of usable context (HA: 52.4 ± 8.0, NA: 52.1 ± 7.5, t(56) = 0.29, p = 0.774) and cue trials (HA: 140.7 ± 15.8, NA: 136.8 ± 19.6, t(56) = 0.69, p = 0.491). Remaining epochs were averaged separately for the three context conditions and the two main phases of the experiment. The averaged epochs were then analysed using the Hilbert transform: data were first submitted to a bandpass-filter (width 0.5 Hz, 12^th^ order Butterworth) around the according driving frequencies of 15 Hz or 20 Hz. The different driving frequencies were chosen to allow disentanglement of the electrocortical responses associated with the central and contextual cues^57^. The time-varying amplitude of the ssVEP signal was then extracted as the modulus of the filtered empirical signal and the Hilbert-transformed analytic signal.

**Figure 2:**
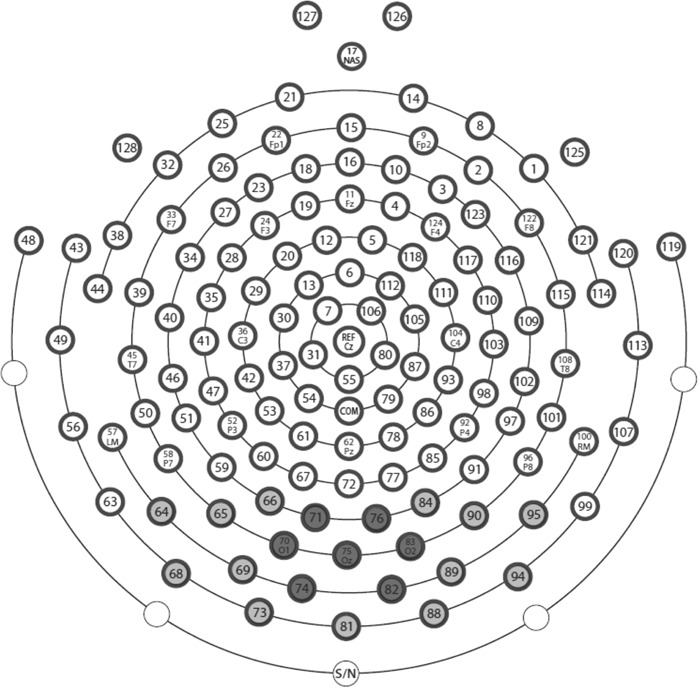
Electrode layout of the 129 electrodes Electrical Geodesics System. The 7 dark grey coloured electrodes were used for central cue ssVEP analyses, whereas all 19 light and dark grey coloured electrodes were used for analyses of contextual cue responses.

For statistical analyses, the ssVEP amplitudes were averaged across time points between 100 ms to 2,900 ms for central cue onset and between 100 ms to 4,900 ms for contextual cue onset. Only the onsets of the contextual cue presentations were analysed, since later time intervals were confounded with electrical stimulations that lead to strong artefacts in the EEG signal. We used the same cluster of electrodes as in our previous study^7^. For central cue analysis, an occipital cluster around Oz and its 6 surrounding sensors was chosen (EGI sensors 70, 71, 74, 75, 76, 82, 83), while a cluster including 20 occipital electrodes (EGI sensors 64, 65, 66, 68, 69, 70, 71, 73, 74, 75, 76, 81, 82, 83, 84, 88, 89, 90, 94, 95) was selected for contextual cue analysis, with the latter covering a larger area, as the peripherally presented contextual cues elicited a more wide-spread electrocortical activation (see Fig. 2).

### Statistical analysis

The mean ssVEP amplitude reactions to central and contextual cues were analysed separately for acquisition and extinction with mixed-measure analysis of variances (ANOVA) with the within-subject factor condition (N *vs* U *vs* P) and the between-subjects factor group (LA *vs* HA). Averaged threat and US-contingency ratings were examined using the same procedure. Significant effects were followed up using *t*-tests. A significance level of 0.05 was used for all analyses and Greenhouse-Geisser correction was applied where appropriate^58^. Throughout this manuscript, the uncorrected degrees of freedom, the corrected *p* values and the partial *η²* (${\eta }_{p}^{2}$) or Cohen’s *d* (*d*) are reported^59^.

## Results

### Central cue analysis

#### Steady-state visual evoked potentials

**Figure 3:**
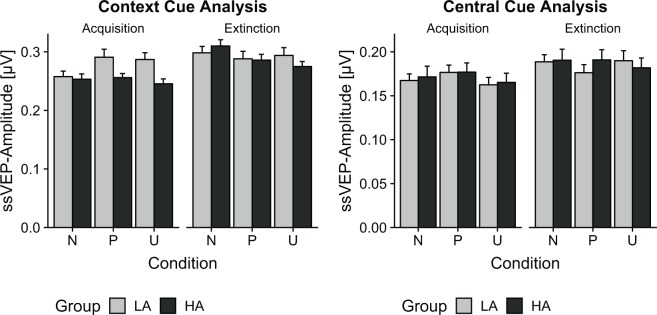
(Left) Mean results (±*SEM*) for central cue ssVEP-analysis (20 Hz) averaged across the whole cue presentation (100–2,900 ms). Central cues in the P-condition elicited highest ssVEP amplitudes during acquisition. (Right) Mean results (±*SEM*) for contextual cue onset ssVEP-analysis (15 Hz) averaged across the first five seconds after contextual cue onset (100–4,900 ms). Only LA individuals show differential processing of the contextual cues during acquisition. From acquisition to extinction, amplitudes to the contextual cues in the N-condition increased.

**Figure 4:**
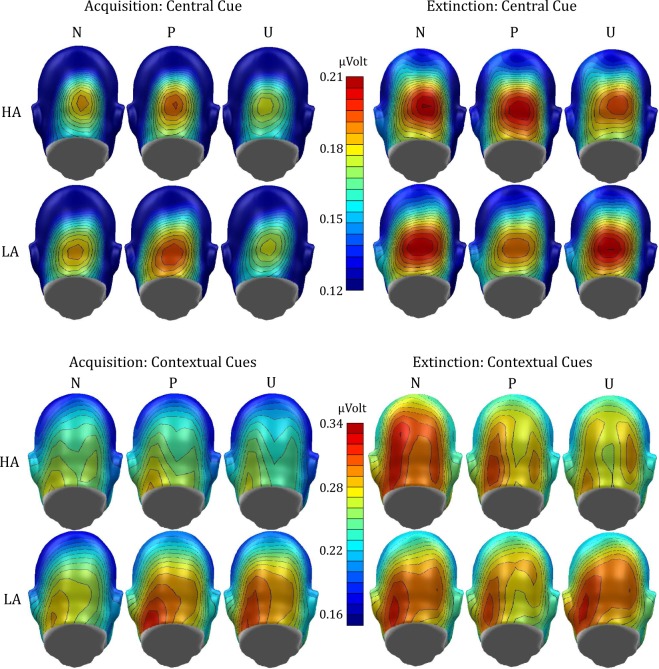
Mean scalp topographies of ssVEP amplitudes to the conditions during acquisition (left) and extinction (right) evoked by the central cue (top rows) and contextual cues (bottom rows).

The ANOVA of the mean ssVEP amplitude in response to the central cues during acquisition revealed a significant main effect of condition, *F*(2,112) = 3.48, *p* = 0.042, ${\eta }_{p}^{2}$ = 0.059. No effects of group reached significance, *p*s > 0.897 (see Figs 3 and 4). Post-hoc paired *t*-tests showed a significant difference between predictable and unpredictable condition, *t*(57) = 2.29, *p = *0.026, *d* = 0.300, but not between predictable and neutral condition, *t*(57) = 1.49, *p = *0.143, *d* = 0.195, nor unpredictable and neutral condition, *t*(57) = 1.45, *p = *0.153, *d* = 0.191. During extinction, no significant effects was found, *p*s > 0.158.

#### Ratings of central and contextual cue combinations

The combination of context and central cues were rated differentially with regard to perceived threat. During acquisition, the ANOVA yielded a significant main effect of condition, *F*(2,112) = 154.71, *p* < 0.001, ${\eta }_{p}^{2}$ = 0.734. The groups did not differ on any level, *p*s > 0.208. As expected, post-hoc *t*-tests revealed that during acquisition cues in the predictable, *t*(57) = 16.64, *p* < 0.001, *d* = 2.184, and in the unpredictable condition, *t*(57) = 11.58, *p* < 0.001, *d* = 1.521, were rated as more threatening than the cues in the neutral condition. Moreover, cues in the predictable condition were rated as more threatening compared to cues in the unpredictable condition, *t*(57) = 4.789, *p < *0.001, *d* = 0.629. The differences between the conditions were still significant during extinction, *F*(2,112) = 42.81, *p < *0.001, ${\eta }_{p}^{2}$ = 0.433, P *vs* N: *t*(57) = 7.53, *p < *0.001, *d* = 0.988, U *vs* N: *t*(57) = 6.77, *p < *0.001, *d* = 0.889, P *vs* U: *t*(57) = 2.02, *p = *0.048, *d* = 0.265, without any effect of group, *ps* > 0.241.

**Figure 5:**
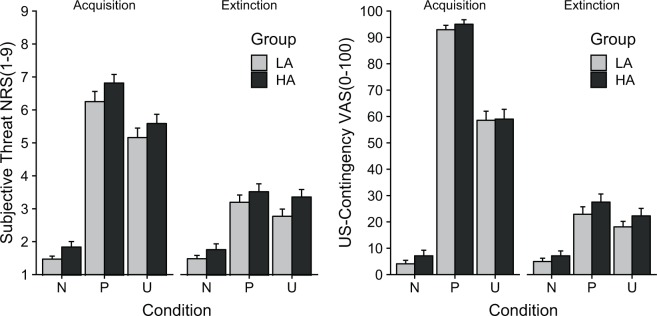
Mean results (±*SEM*) for subjective threat (left) and US-contingency (right) ratings for central cue analysis. During acquisition the combination of central and contextual cues in the P-condition are rated with highest subjective threat and US-contingency. No differences were found between groups.

As for threat ratings, the ANOVA for US-contingency during acquisition showed a significant main effect of condition, *F*(2,112) = 283.22, *p < *0.001, ${\eta }_{p}^{2}$ = 0.835, without any effects of group, *p*s > 0.463 (see Fig. 5). Again, post-hoc *t*-tests showed that cues in the predictable, *t*(57) = 30.56, *p < *0.001, *d* = 4.012 and in the unpredictable condition, *t*(57) = 11.93, *p < *0.001, *d* = 1.567, were associated with higher shock expectancy than in the neutral condition. While the cues in the predictable condition had also higher likelihoods than the cues in the unpredictable condition, *t*(57) = 9.75, *p < *0.001, *d* = 1.281. During extinction, the differences between conditions stayed significant, *F*(2,112) = 34.94, *p < *0.001, ${\eta }_{p}^{2}$ = 0.384, P *vs* N: *t*(57) = 6.63, *p < *0.001, *d* = 0.870, U *vs* N: *t*(57) = 5.99, *p < *0.001, *d* = 0.786, P *vs* U: *t*(57) = 3.01, *p = *0.004, *d* = 0.395. No effect of group could be observed, *ps* > 0.359.

### Contextual cue analysis

#### Steady-state visual evoked potentials

The ANOVA of the mean ssVEP amplitudes in response to the contextual cues during acquisition yielded a significant main effect of condition, *F*(2,112) = 3.57, *p = *0.032, ${\eta }_{p}^{2}$ = 0.060, that was further qualified by a significant Group x Condition interaction, *F*(2,112) = 4.23, *p = *0.017, ${\eta }_{p}^{2}$ = 0.070 (see Figs 3 and 4). The main effect of group did not reach significance, *F*(1,56) = 2.07, *p = *0.156, ${\eta }_{p}^{2}$ = 0.036. To follow-up on the interaction, separate ANOVAs were first calculated for groups revealing a significant main effect of condition in the low anxious group, *F*(2,56) = 5.99, *p = *0.004, ${\eta }_{p}^{2}$ = 0.176, but not in the high anxious group, *F*(2,56) = 0.82, *p = *0.446, ${\eta }_{p}^{2}$ = 0.028. For low anxious participants, the predictable, *t*(28) = 3.30, *p = *0.005, *d* = 0.563, and unpredictable, *t*(28) = 2.95, *p = *0.006, *d* = 0.548, contextual cues elicited stronger electrocortical responses than the neutral contextual cues, whereas no difference was found between the predictable and unpredictable condition, *t*(28) = 0.37, *p = *0.713, *d* = 0.069. Subsequently, directed comparisons of the groups showed that low anxious individuals responded to the unpredictable condition with larger ssVEP amplitude than high anxious individuals, *t*(56) = 2.05, *p* = 0.046, *d* = 0.537. During extinction, a main effect of condition was found, *F*(2,112) = 3.87, *p = *0.024, ${\eta }_{p}^{2}$ = 0.065, without any effect of group, *p*s > 0.149. Electrocortical responses to the neutral contextual cues were stronger than to the predictable and the unpredictable contextual cues, *t*(57) = 2.27, *p = *0.027, *d* = 0.298 and *t*(57) = 2.60, *p = *0.012, *d* = 0.342. No difference was found between predictable and unpredictable contextual cues, *t*(57) = 0.30, *p = *0.763, *d* = 0.040.

#### Ratings of contextual cues

**Figure 6:**
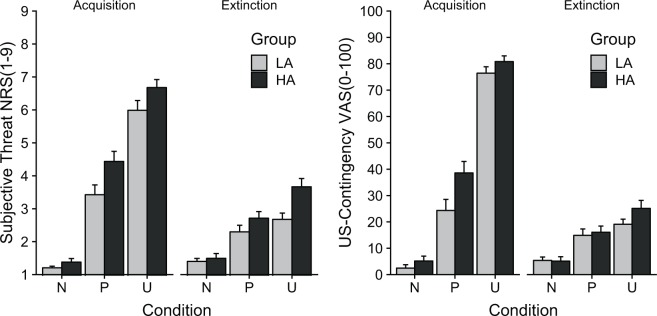
Mean results (±*SEM*) for subjective threat (left) and US-contingency (right) ratings for contextual cue analysis. During acquisition the contextual cues in the U-condition were rated with highest subjective threat and US-contingency. HA individuals demonstrated higher threat ratings in general than LA individuals.

The ANOVA of the perceived threat ratings during acquisition yielded a significant main effect of condition*, F*(2,112) = 134.79, *p < *0.001, ${\eta }_{p}^{2}$ = 0.706. In addition, a marginal significant main effect of group was found, *F*(1,56) = 4.59, *p = *0.055, ${\eta }_{p}^{2}$ = 0.064. However, the Group x Condition interaction did not reach significance, *F*(2,112) = 0.95, *p = *0.387, ${\eta }_{p}^{2}$ = 0.017 (see Fig. 6). The high anxious group generally rated contextual cues as more threatening compared to the low anxious group. To follow up on the main effect of condition, several *t*-tests were calculated. The unpredictable context was rated with higher threat ratings compared to the neutral and predictable context, *t*(57) = 18.14, *p < *0.001, *d* = 2.381 and *t*(57) = 7.34, *p < *0.001, *d* = 0.964. The predictable context was perceived as more threatening than the neutral context as well, *t*(57) = 8.41, *p < *0.001, *d* = 1.104. During extinction, these differences remained significant, *F*(2,112) = 35.76, *p < *0.001, ${\eta }_{p}^{2}$ = 0.390, P *vs* N: *t*(57) = 5.06, *p < *0.001, *d* = 0.664, U *vs* N: *t*(57) = 7.97, *p < *0.001, *d* = 1.046, P *vs* U: *t*(57) = 3.36, *p < *0.001, *d* = 0.441. Moreover, the ANOVA yielded a marginal significant main effect of group, *F*(1,56) = 2.99, *p = *0.089, ${\eta }_{p}^{2}$ = 0.051, and a marginal interaction effect between group and condition, *F*(2,112) = 2.44, *p = *0.092, ${\eta }_{p}^{2}$ = 0.042. Exploratory follow-up tests revealed a significant difference in the unpredictable condition during extinction between high vs. low anxious individuals, *t*(56) = 2.28, *p* = 0.027, *d* = 0.559, indicating higher threat ratings to the unpredictable context during extinction for high anxious individuals.

Analysis of US-contingency during acquisition showed a main effect of condition, *F*(2,112) = 154.49, *p < *0.001, ${\eta }_{p}^{2}$ = 0.734, and a significant main effect of group, *F*(1,56) = 4.95, *p = *0.030, ${\eta }_{p}^{2}$ = 0.081, but not their interaction, *F*(2,112) = 1.05, *p = *0.338, ${\eta }_{p}^{2}$ = 0.018 (see Fig. 6). Post-hoc *t*-tests revealed significant differences between all conditions, U > N: *t*(57) = 25.90, *p < *0.001, *d* = 3.401, U > P: *t*(57) = 9.14, *p < *0.001, *d* = 1.199, P > N: *t*(57) = 6.09, *p < *0.001, *d* = 0.779, with overall higher US-contingency ratings for high anxious individuals. During extinction, the condition effects remained significant, *F*(2,112) = 24.05, *p < *0.001, ${\eta }_{p}^{2}$ = 0.300, U > N: *t*(57) = 7.07, *p < *0.001, *d* = 0.928, U > P: *t*(57) = 2.59, *p = *0.012, *d* = 0.340, P > N: *t*(57) = 4.28, *p < *0.001, *d* = 0.562, without any effect of group, *ps* > 0.408.

## Discussion

The purpose of the present study was to compare electrocortical correlates of attention allocation during predictable and unpredictable threat acquisition and extinction learning in high vs. low anxious individuals. For this goal, we investigated steady-state visual evoked potentials along evaluative ratings in a combined cue and context conditioning paradigm.

Ratings of threat and US-contingency demonstrated that predictable and unpredictable threat was acquired as expected. Both groups showed elevated evaluative responses to the central cue in the predictable threat condition and to contextual cues in the unpredictable threat condition, replicating our earlier results^7^. Moreover, verbal responses to predictable and unpredictable threat cues decreased during extinction.

Low and high anxious individuals did not differ regarding ratings related to predictable threat, but during acquisition, high anxious individuals demonstrated higher threat and contingency ratings for contextual cues than low anxious participants irrespectively from the condition. These results suggest elevated sensitivity to contextual threat in high anxious individuals and are in line with a growing body of literature suggesting that heightened sensitivity to unpredictable threat may be a hallmark for anxiety disorders^21–23^ and extend these effects to a subclinical sample. Post-hoc analyses during the extinction phase revealed higher threat ratings in the unpredictable threat condition for high anxious compared to low anxious individuals, which is in line with a recent meta-analysis, indicating reduced or slowed extinction learning in patients with anxiety disorders^46,60^. Even though the meta-analytical results are mainly based on predictable threat conditioning paradigms, in our experiment high trait-anxious individuals demonstrated reduced extinction learning for unpredictable threat only. This lends further support for the notion that high anxious individuals are especially sensitive to uncertain threat.

During predictable threat conditioning, sensory processing was facilitated for the central cue in the P-condition compared to the U-condition, supporting the idea of selective attention to specific threat stimuli^8,9,31^. However, in contrast to our earlier study, we only found a descriptive, non-significant difference between the P- and the N-condition, which may be due to methodological variations in the present design.

Regarding contextual cue onset analysis, different patterns were observed for high and low anxious individuals. Low anxious individuals showed increased activity for the unpredictable and predictable threat contexts compared to the neutral context. This confirms results of Kastner-Dorn *et al*.^8^. Thus, contextual cues, associated with an aversive event - independent of threat predictability - draw more attentional resources than the safety context. This result supports the notion that threat relevant stimuli gain enhanced visual processing. After all, the onset of the predictable and unpredictable contextual cue both signal aversive events during the next 32 seconds, while only the neutral context reliably signals no upcoming aversive events. During the predictable context, however, aversive events are further signalled by a discrete cue, but not in the unpredictable context. Paradoxically, the onset of the discrete cue during the predictable condition is unpredictable, which in turn serves as a source of uncertainty about when the aversive event appears. Note that Wieser *et al*.^7^ found enhanced visual processing of the unpredictable contextual cues only. However, in contrast to this previous study, we this time analysed not only the first second but an interval of five seconds after contextual cue onset. In the same way, other studies report that during the first second after stimulus onset the ssVEPs does not discriminate as strongly between CS+ and CS− as in a later interval^35,61,62^, which could explain the found discrepancies.

High anxious individuals did not differentiate in ssVEP activity to the contextual cues among conditions. A very similar pattern was recently observed in a study by Ahrens *et al*.^43^ who associated different neutral faces with neutral, positive or negative verbal comments. In contrast to low socially anxious individuals, high socially anxious individuals did not differentiate between the three conditions in the ssVEP amplitudes, indicating a potentially impaired ability to discriminate between relevant and irrelevant social stimuli. The authors interpreted this finding as a possible hyperactivation of the amygdala and a consequent overgeneralization of conditioned social threat in social anxious individuals. In the current study, high anxious individuals might similarly be characterised by a discrimination deficit for contextual cues, explaining the diminished visual discrimination of these cues. In addition, high anxious individuals also showed attenuated attentional processing of the threatening contextual cues compared to low anxious individuals, which might suggest a potential indicator of perceptual avoidance in early stages of visuocortical processing. Even though there is a vast body of literature describing attentional hypervigilance-avoidance biases in anxiety^63^, empirical evidence for perceptual avoidance in ssVEP measures is sparse^44^. Notably, a recent study observed reduced ssVEP amplitudes to aversive relative to neutral facial expressions for severely impaired social anxiety patients^64^. In this study, visuocortical responding to threatening stimuli in anxiety patients depended on the severity of the disorder: patients diagnosed with circumscribed social anxiety showed increased vigilance to aversive facial expressions, while attentional processing of the most impaired patients was actually decreased, reflecting a possible indicator of perceptual avoidance^64^. Although, we investigated solely high anxious, subclinical individuals, accumulating evidence indicates that initial hypervigilance and consequent perceptual avoidance of aversive stimuli might be a potential risk factor characterizing highly anxious individuals^65^. However, it should be noted that due to early non-stationary components of ssVEPs^35,37^, it might be difficult to capture the initial short-lasting hypervigilance, which is already evident in early electrocortical components (about 50–100 ms) of visual event-related potentials^66,67^. For that reason, it is important to mention, that we cannot definitely distinguish between impaired discrimination abilities or perceptual avoidance in high anxious individuals. Both mechanisms seem plausible. Also, they are not necessarily mutually exclusive and could occur together. Future research may address this issue by using a combination of eye tracking and ssVEPs to record measures of initial hypervigilance and sustained visuocortical responding within the same paradigm. Further, these sensory processing biases could also explain the elevated threat and contingency ratings for the contextual cues independent of the conditions. Since high anxious individuals might be worse at discriminating or perceptually avoid contextual threat stimuli, they accordingly overestimate the probability and aversiveness of unpredictable threat.

During extinction learning, only the context which indicated safety in the acquisition phase gained enhanced attentional resources. A reason for that could be that participants realized the sudden absence of aversive events and consequently grew suspicious of the other conditions, since the neutral condition may have become the best candidate for a change in the US-contingencies. Accordingly, the increased visuocortical processing of the former neutral stimuli may reflect the electrocortical correlate of an attentional reorientation process. A very similar pattern was found in McTeague *et al*.^42^, where visuocortical responses to former safety stimuli increased and responses to former threat stimuli decreased during extinction. The reason why responses to the former threatening stimuli did not decrease during extinction seems in contrast to the results of McTeague *et al*.^42^. However, it should be considered that we instructed participants only in regard to the acquisition phase, but not about the absence of the US during extinction. Also, another explanation for the increased ssVEP amplitudes to the neutral contextual cues during extinction learning seems plausible: As the neutral contextual cues predict – and have always predicted - absence of shocks, they stay the last reliable predictor during extinction learning. Stimuli with a strong predictive value usually are also motivationally significant, which in turn is associated with stronger visuocortical processing^31^. To elucidate potential underlying mechanisms, a recent steady-state visual evoked field (ssVEF) study used a predictive modelling approach to explain increased ssVEF amplitudes to the conditioned safety stimulus during extinction learning^68^. The authors showed that changes in visuocortical processing reflect the associative strength of the CS^69^ and can be predicted by estimates of the Rescorla-Wagner model^70^.

One limitation of our current study is that we could only analyse the onset of the contextual cue presentation, since later time intervals were confounded with electrical stimulations that lead to strong artefacts in the EEG signal. Consequently, we can make no statements about visuocortical processing of the contexts during the whole trial. Even so attentional mechanisms seem to be stable over time^8,71^, differences between low and high anxious individuals as well as extinction processes should be considered throughout the whole context presentation intervals in future studies. Finally, since the central cue during the unpredictable condition gained salience, though it was never directly associated with an US, the question arises, whether threat cues are differently processed in contexts that were associated with aversive events compared to contexts that are perceived as safe. In the NPU-threat task, predictable and unpredictable threat are fully separated by conditions, making it a perfect tool to simultaneously investigate fear- and anxiety-like processes in the laboratory. However, it might be important to consider an interplay between cue and context conditioning and hence a possible interaction between fear and anxiety^72^. Future studies may also test if contextual discrimination deficits contribute to a poorer treatment response in anxiety disorders^73^ and whether they can be targeted with attentional discrimination training^74^ to potentially improve the treatment of anxiety disorders.

Altogether, our results support the idea that individuals at risk for anxiety disorders are characterized by aberrant processing of unpredictable threat cues^21,23^. In line with recent findings, attention is selectively focused onto the specific threat stimulus during predictable threat, while during unpredictable threat attentional vigilance for the context is initially increased for healthy low anxious individuals^7,8^. On the contrary and strikingly, high anxious individuals are less able to discriminate between aversive contexts and might even perceptually avoid threatening relative to safe contexts on an early visuocortical level. Increased visual processing of former safety stimuli during extinction learning may reflect electrocortical correlates of an active attention reorientation process after the change of contingencies^42^.

## Data Availability

The datasets generated during and/or analysed during the current study are available from the corresponding author on reasonable request.
